# Pharmacy-based needle exchange in New Zealand: a review of services

**DOI:** 10.1186/1477-7517-2-10

**Published:** 2005-07-12

**Authors:** Janie Sheridan, Charles Henderson, Nicola Greenhill, Andrew Smith

**Affiliations:** 1 School of Pharmacy, University of Auckland, 85 Park Road, Grafton, Auckland, New Zealand; 2 National Manager, Needle Exchange Programme New Zealand, 172 Manchester Street, Christchurch, New Zealand; 3 Pharmacy Department, Derriford Hospital, Plymouth, UK; 4 Northumbria Healthcare NHS Trust, UK

## Abstract

**Background:**

New Zealand has been offering needle exchange services since 1987. Over 170 community pharmacies are involved in the provision of this service. However, no recent detailed review of New Zealand's pharmacy-based needle exchange has been published. This study aimed to explore service provision, identify problems faced by pharmacists, and look for improvements to services.

**Methods:**

The study used a cross-sectional survey of all needle exchange pharmacies. Postal questionnaires were used with postal and telephone follow-up.

**Results:**

A response rate of 88% was obtained overall. Pharmacists had been providing the service for a mean of 6 years. Pharmacies had given out an average of 130 injecting units, in a mean of 62 transactions to a mean of 17 clients in the 4 weeks prior to completing the questionnaire. The majority had not incurred problems such as violence or intoxicated clients in the last 12 months, although almost one third had experienced shoplifting which they associated with service provision. Training and improving return rates were identified as potential areas for further development.

**Conclusion:**

New Zealand needle exchange pharmacies are providing services to a number of clients. The majority of service providers had been involved for a number of years, indicating the problems incurred had not caused them to withdraw their services – findings which echo those from the UK. Further training and support, including an exploration of improving return rates may be needed in the future.

## Background

During the 1980s with the advent of HIV and the realisation that the virus could be spread through shared, contaminated injecting equipment, a number of countries set up needle exchange programmes. These have been defined as services provided for the exchange of sterile injecting equipment for used injecting equipment, as a potential means of reducing the transmission of infectious diseases. They may operate as 'stand alone' agencies, from mobile outlets, in accident and emergency units at hospitals, from drug treatment services and from community pharmacies.

Needle exchange services began to be offered in New Zealand in May 1987 [1]. Currently, New Zealand's needle exchange activities are enabled by the New Zealand Ministry of Health (MoH) contracting drug user groups, constituted as charitable trusts, to run individual needle exchanges as separate entities (known as dedicated exchanges) and operating under a peer service model. There are 12 full-time dedicated needle exchanges, two part-time, and a trial regional mobile service on the West Coast of the South Island. In addition, over 170 community pharmacies (retail pharmacies) out of approximately 900 provide needle exchange services. Pharmacy-based services either operate at Level 1 (only needle and syringe packs with condom, lubricant, alcohol swabs, educational material and personal sharps container) or Level 2 (can provide single needle and syringe sales and other harm reduction equipment sales as well as Level 1 'packs'). Additionally the MoH funds the operation of a national office to (a) operate collection and destruction service for sharps waste generated by the needle exchange programme (NEP) and (b) to generally co-ordinate, liaise and disseminate information between stakeholders and NEP service providers – both nationally and internationally.

In a survey of all needle exchanges in the UK, it was estimated that 27 million syringes were distributed annually in 1997, with community pharmacies distributing an equal number of syringes as non-pharmacy outlets; however, non-pharmacy outlets were visited more frequently [2]. In New Zealand, around one million injecting units are distributed annually and this figure has remained constant for the last three years, with approximately 75% of total volume of injecting equipment being provided via dedicated exchanges, the remainder from pharmacy based outlets (personal communication).

A number of reasons have been suggested for clients preferring either to go to pharmacy needle exchanges or stand alone services. In an Australian study of pharmacy-based and agency needle exchanges, client characteristics were found to be similar in terms of demographics and health problems. Proportions of both groups indicated that they used both types of exchange facilities [3]. A study of clients from pharmacy needle exchanges in London found that clients who indicated overall they preferred going to a drug agency needle exchange, rated them more highly on issues such as range of equipment available, being 'no hassle' and the staff being sympathetic. Those who preferred pharmacy needle exchanges rated the level of confidentiality more highly, along with ease of access and being open when needed [4].

The involvement of community pharmacy in needle exchange in New Zealand (personal communication), as in England and Wales [5], is around 1 in 5. However, unlike in the UK, until late 2004, pharmacy-based needle exchange in New Zealand operated under a 'user pays' system and no remuneration was provided directly to pharmacies. Pharmacies therefore covered their costs through profits on the sales of injecting equipment. However, recently, a free one-for-one service has been set up which provides 3 ml barrels and all injecting needles (excluding butterflies and piercing needles) free to clients who return a used syringe. Other equipment remains available under the 'user-pays' system, with returns containers provided for free to encourage returns.

Although many community pharmacies provide needle exchange, there are many pharmacists who are reluctant to engage in this service provision, citing reasons such as lack of time and space, previous bad experiences and client behaviour [5-7]. A study of South East England needle exchange pharmacies found that pharmacists providing needle exchange did experience problems such as shoplifting and intoxicated clients disrupting the pharmacy, but that more serious problems such as violence were vary rare [8].

The first year of operation of needle exchanges in New Zealand has been described by Lungley and Baker [9], and more recently reviewed by Kemp and Aitken [1]. However, despite the existence of pharmacy-based needle exchanges in New Zealand since the late 1980s, very little information exists about their operation and the issues faced by pharmacists. Community pharmacies form an important part of the overall national needle exchange programme, and it is essential that issues facing service providers are monitored and managed. Studies of the activities of pharmacy-based needle exchange in the UK have uncovered significant issues such as the need for training, information materials, and effective and efficient support services [7,8].

The aims of this study were to:

• describe current practice with regard to the provision of needle exchange;

• estimate the level of service provision;

• explore issues and problems with regard to service provision;

• identify areas for improvement in the programme.

The study used a methodology and questionnaire based on similar research conducted by JS in South East London [8].

## Methods

The study employed a cross sectional survey design, using a self-completion postal questionnaire with postal and telephone follow-up. All community pharmacies listed by Needle Exchange Services Trust (NEST) as providing a needle exchange service were included in the sample (N = 176). The study was carried out between June and August 2003, at a time when all pharmacy needle exchanges were still operating under a 'user-pay' system.

The questionnaire was based on one successfully utilised in England [8] and adapted to suit a New Zealand context. The questionnaire was designed to collect data on a number of areas of service provision, demographics of the pharmacy and pharmacist, levels of activity within the needle exchange, services provided to needle exchange clients, other services provided to drug misusers, problems and conflicts with service provision and potential improvements to the service.

The New Zealand version of the questionnaire was piloted among a group of key informants (who were not currently working in any of the needle exchange pharmacies, but who had knowledge of the scheme and/or prior experience). Modifications based on results of the pilot were made to the questionnaire which was to be administered by post. A shorter version of the questionnaire was devised using key questions from the postal questionnaire and used to follow up non-responders to the two mailshots by telephone.

Questionnaires and Participant Information Sheets were mailed to all pharmacies listed as providing needle exchange during June 2003. Each questionnaire contained an ID number so that responders could be noted in a database. After three weeks, non-responders were sent a reminder letter and another copy of the questionnaire. After another 3 weeks, remaining non-responders were contacted by telephone and asked if they would be willing to complete a shorter version of the questionnaire over the telephone.

Data were entered into SPSS^® ^(a statistical database package), and analysed using appropriate descriptive statistics. Further analyses were undertaken looking at differences between groups using appropriate parametric and non-parametric statistics.

Approval to conduct this study was obtained from the University of Auckland Human Participants Ethics Committee.

## Results

Of the 176 pharmacies listed by NEST, usable responses were obtained from 153. Information received indicated that a further one pharmacy had closed, two no longer considered themselves part of the scheme and one could not be contacted. One hundred and sixteen (67.1%) responded to the self-completion postal questionnaire and the remaining 37 responded to the telephone questionnaire (thus providing data on a limited number of questions). The final response rate was thus 88.4% (153/173).

Unless otherwise stated, results pertain to the total respondent group (i.e. responses from the two mailshots and the telephone follow-up).

### Respondent demographics

Respondents had been working in community pharmacy for a mean of 23.0 years (sd = 11.6; range = 12–51 years) and at that particular pharmacy for a mean of 13.7 years (sd = 10.3; range = 5 months-44 years). They had been part of the needle exchange programme at that particular pharmacy for a mean of 6.0 years (sd = 4.3; range = 1 month – 18 years). Sixty five percent were male. Respondents described themselves as being located in city/town centre (22.9%), suburban area of large town or city (40.5%) or small town/township servicing rural hinterland (36.6%). In relation to other shops or businesses, location of premises was described as: main shopping street (51.6%); indoor shopping mall (7.2%); small group of local shops (32.7%); health centre (10.5%) and 'other' (2.6%) (adds up to >100% as respondents could tick more than one box). Just over half (54.2%) of pharmacies were part of a Banner group (franchise).

The majority were full-time pharmacists (61.4%) and pharmacy owners (63.4%) with just over one quarter (27.5%) classifying themselves as a pharmacist manager. The remaining options were locum pharmacist (2.0%), employee pharmacist (3.9%), regular part-time pharmacist (5.2%) and other (non-pharmacist) (1.4%) (adds up to >100% as respondents could tick more than one option).

Respondents were asked to indicate why they became part of the scheme by ticking options from a list (respondents could tick more than one option) (mailshots 1–2 only). The most commonly chosen options were "to protect the community from needle-stick injuries" (81.0%), "reduce New Zealand healthcare costs" (53.4%) and "regard it part of being a health professional" (81.9%). Very few chose the option "profitability/ business reasons" (6.9%). Additional reasons cited for involvement were to reduce spread of blood borne viruses (7); harm reduction (3); reduce local crime (1); protect pharmacy against crime (1); family experience of drug misuse (1); provide a local service (1) and protect local community (1).

### Services provided as part of the scheme

The majority of pharmacies (57.5%) were involved in the provision of level 2 services (see Introduction) (data missing on one case).

Respondents were asked to estimate needle exchange activity in the four weeks prior to completing the questionnaire. Table 1 provides data from these responses. Nineteen pharmacies (12.4%) had not conducted any needle exchange transactions during this time and just over one fifth (20.9%) said they had no regular clients (defined as having attended about once a month or more frequently). On all four measures, level 2 needle exchange pharmacies had a significantly higher service activity than those providing Level 1.

**Table 1:** Data on needle exchange activity

		N	Mean (sd)	Median	Min	Max	MW-U; p = (data missing on 1 case)
How many NX interactions took place in the last 4 weeks?	Total	149	62.4 (128.7)	12	0	840	943; p < 0.0001
	L1	63	26.5 (107.1)	3	0	800	
	L2	85	89.8 (137.6)	40	0	840	
How many different clients used service in last 4 weeks?	Total	107	17.0 (35.7)	6	0	250	495.5; p < 0.0001
	L1	41	6.2 (16.6)	2	0	100	
	L2	65	24.1 (42.6)	10	0	250	
How many clients use the service regularly?	Total	133	11.6 (23.4)	5	0	200	821; p < 0.0001
	L1	54	4.6 (14.0)	1	0	100	
	L2	78	16.6 (27.3)	9	0	200	
How many individual injecting units were issued in the last four weeks?^1,2^	Total	99	130.0 (195.9)	50	0	1200	480.5; p < 0.0001
	L1	32	65.7 (156.5)	20	0	800	
	L2	66	163.2 (206.9)	85	0	1200	

1. Defined as sufficient injecting equipment for one injecting, e.g. one syringe, or one needle plus barrel

2. Mailshots 1–2 only

L1 and L2 = level 1 and level 2 needle exchange

As well as verbal information to clients, pharmacists have the ability to provide, as part of their distribution activities, educational leaflets on matters related to needle exchange. The Health [Needles & Syringes] Regulations 1998 that govern the authorised sale of needles and syringes in New Zealand state that all sales of injecting equipment in New Zealand must be accompanied by some educational material. Table 2 shows the proportions of respondents indicating (by ticking a box for 'yes') that they had leaflets on specific topics in the pharmacy. With the exception of leaflets on hepatitis B, over 44% had leaflets on related subjects such as safer sex, safer injecting and testing for HIV. The most commonly stocked leaflet was one on other needle exchange outlets (including contact address and phone number).

**Table 2:** Leaflets in the pharmacy (N = 116 – mailshots 1–2 only)

Leaflet type	Those ticking 'yes' N (%)
Safer sex	58 (50.0)
Safer injecting	59 (50.9)
Testing for HIV/hepatitis	55 (44.7)
Hepatitis C information	60 (51.7)
Hepatitis B information	32 (27.6)
Needle exchange outlets	81 (69.8)

Pharmacists were asked whether NES clients made use of other related services provided by the pharmacy, ticking a box for 'yes' (mailshots 1–2 only) and included: dispensing prescriptions for methadone substitution therapy (48.3%), dispensing prescriptions for other Controlled Drugs e.g. benzodiazepines (37.9%), providing written advice on safer drug use (19.0%), verbal advice on safer drug use (14.7%), advice on hepatitis testing (5.2%), advice on HIV testing (4.3%), advice on safer sex (6.0%) and leaflets in non-English (6.0%).

### Service policies and procedures

Although in many cases it is the pharmacist who conducts needle exchange transactions, a trained member of staff may also do so. Only five respondents (4.2%) indicated that only 'specially designated staff' would undertake needle exchange transactions, around one third (36.2%) indicated that it would be only the pharmacist, and just over one quarter (27.5%) indicated that it would be pharmacists and staff who felt comfortable in the role. The most common response was "all staff" (42.2%) (mailshots 1–2 only) (adds up to >100% as respondents could tick more than one option).

One important part of needle exchange is that injecting equipment is returned to a needle exchange outlet for safe disposal; (this may not always be the same outlet as the supplying outlet). Respondents were asked what their 'policy' was around supply and return of equipment. Almost 3% stated they supplied strictly on a "one for one" basis, 19.6% said they strongly encouraged returns, 45.8% said they encouraged returns, with over one quarter (28.1%), reporting that returns were not pursued (the remainder said "other") (data missing on 4 cases). There was no significant difference in returns policy between levels 1 and 2.

When asked about limits on the amount of equipment given out in one transaction, only four respondents said they had limits (data missing on 2 cases). Where stated, the limit was usually 10 injecting units (NB: An injecting unit is equipment needed for one injection, for example: one complete syringe; one barrel and one needle or one barrel and one butterfly).

When asked what the pharmacy policy was for clients owing money for equipment, 49.7% said there was no credit under any circumstances, 21.6% said they decided on a case-by-case basis, one person gave credit to anyone who requested it, the remainder stating "other". Only one person gave an indication of the credit limit, which in that case was $NZ10 (mailshots 1–2 only).

Respondents were asked to indicate what encouraged clients to ask for help. Almost 89.7% indicated "attitude of staff". Almost two thirds (62.1%) indicated that a client being a regular user of the service was important, but less than one third (30.2%) stated "staff being pro-active".

### Support for pharmacists

**Figure 1:**
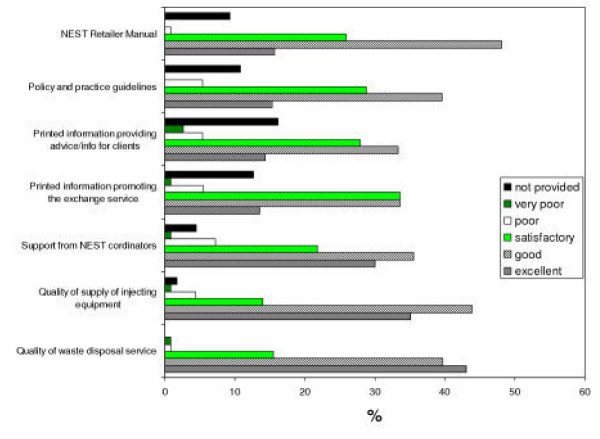
Satisfaction with support services (N = 116; mailshots 1–2 only).

In order to provide services, a number of support systems need to be in place. These include supply of sterile injecting equipment to pharmacies, collection of waste materials, training, and leaflets. Respondents were asked to rate the quality of this support. Figure 1 shows the results. Where provided, most services were considered to be at least satisfactory, although around 10% felt that promotional information and printed advice for clients was poor. Furthermore, a small proportion (8%) stated that support from NEST co-ordinators was poor, and 4.5% indicated it was not available. Significant numbers reported that they did not have a copy of the NEST Retailer Manual, Policies and Guidelines and printed advice to give to clients, although 87% had stated that they had read the Retailer Manual.

### Training

Respondents to mailshots 1–2 were asked to indicate training received by ticking a box for 'yes'. Just over one quarter (26.7%) had attended training sessions, 59.5% had received written training materials, 27.6% stated they had received no training and one person 'didn't know' (% add up to more than 100% as some people may have received written materials and also undertaken a training session).

Half the respondents stated they were either 'very satisfied' or 'satisfied' with the training, with only 10% being either 'dissatisfied' or 'very dissatisfied' (data missing on 14 cases). Suggestions for further training included provision of videos, recent information updates, explanation of injection equipment and its uses, drug use terminology, training for new staff, provision of printed training materials and pamphlets, dealing with difficult situations and information on returned equipment.

### Problems and difficulties

**Figure 2:**
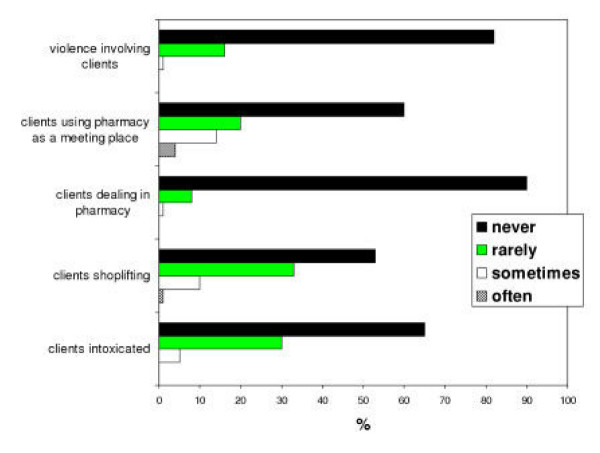
Frequency of problems in the last 12 months (N = 116).

Respondents in mailshots 1–2 were asked to estimate the frequency of certain 'problems' relating to the provision of needle exchange over the 12 months period prior to completing the questionnaire. Data were excluded on those who had not worked at the pharmacy for at least 12 months. Results are shown in Figure 2. Serious problems such as violence were extremely rare occurrences with respondents indicating that in 85% of cases, this had never occurred during the time period studied. Other problems such as shoplifting and clients intoxicated and upsetting other customers occurred at least 'rarely' during this period in 45.1% and 32.8% of cases respectively. When asked how such occurrences were dealt with, respondents reported: calling the police, telling clients their behaviour was unacceptable, giving them a warning or dealing with each occurrence on a case-by-case basis.

When asked whether they had refused to carry out a needle exchange transaction during the twelve month period, 5% said they had refused to supply an under-16 year-old (mailshots 1–2 only), 17% had refused a disruptive client (mailshots 1–2 only), 8% had refused a shoplifting client (mailshots 1–2 only) and 4% had refused a client who was also on an oral methadone prescription (data missing on 3 cases in all above). None reported refusing to engage in a needle exchange transaction with clients who had no used equipment to return to the pharmacy (mailshots 1–2 only) (data missing on 1 case).

In order to assess whether providing the needle exchange service might impact on other customers, respondents were asked to indicate what they thought were their customers' views on the scheme, by ticking a box for 'yes' where applicable. Nineteen percent indicated that their customers viewed these services 'favourably', 11.2% 'unfavourably, and 81.9% indicated that they thought customers were unaware of the scheme (adds up to >100% as respondents could tick more than one option).

### Improving the service

Respondents were asked an open question for suggestions on how the needle exchange service could be improved. Suggestions included improving returns rates (including providing incentives for returns to providers and clients), provision of additional training, advertising the service, cheaper injecting equipment, moving to a free one-for-one service, all equipment being free to clients, involvement of more pharmacies, improved stock supply and information from suppliers, more time to provide advice, provision of a private consultation area, support from specialist agencies, leaflets on issues such as 'coming off drugs', improving returns rates, referral to treatment (including referral information which can be put into packs) and reducing fear around police attitudes towards the service.

## Discussion

This study is the first published, in-depth review of pharmacy-based needle exchange in New Zealand in the last 10 years. The methods used obtained an extremely high response rate – 67% to the postal questionnaire and almost 90% overall including the telephone follow-up. In general, response rates of over 70% are considered to be acceptable in order to generalise to the whole population. The review was extensive covering areas of practice, service delivery levels, areas of conflict, support and training, and ways of improving the service. Pharmacists in this study had been qualified for a number of years and had been involved in the Needle Exchange programme for a mean of 6 years, and were therefore providing feedback to the study from a position of experience. However, it should be noted that pharmacists who had previously provided the service, but were no longer part of the scheme, were not included, and their attitudes and experiences may well be different to those in the study, in particular in relation to experiencing problematic situations.

The level of activity ranged from no involvement in the previous four weeks by one fifth of pharmacies, to high levels of transactions (one fifth undertook 100 or more transactions during this period). Further investigation of those who had been 'dormant' during the study period needs to be undertaken, with regard to location of the outlet, need for the service in that area and whether relocation of the service to a more appropriate outlet might be more viable.

Similar variations were noted in 'number of clients' and 'number of regular clients'. Results from this study indicate the many participating pharmacies were providing a service to a number of clients who attended the pharmacy on a regular basis. This provides opportunities for further intervention if appropriate, such as referral for treatment, health care and social support and are consistent with findings in the UK [8].

A large proportion of pharmacies offered leaflets on a number of related areas such as HIV and hepatitis testing, safer injecting and safer sex, although it appears that clients do not avail themselves of this service very often. One reason might be that clients are not made aware of the presence of these leaflets. Secondly, if aware of them, they may not wish to pick them up for fear of being 'exposed' as drug users. Thirdly they may not feel they need them. Further research needs to be undertaken into the appropriateness of the leaflets and their location, and clients' views and needs with regard to information provision in this manner. From a pharmacy perspective, a lack of private area and training have been identified as being barriers to greater involvement in information provision [10].

Training is an essential component of service delivery. Whilst pharmacists may be willing to provide services, it is unlikely they will have a detailed understanding of many of the issues around injecting. Furthermore, for services such as needle exchange there may be issues around stigmatisation, practitioner attitudes, or staff reluctance to provide services. Results indicated that training is an area where further development may need to take place. One quarter of respondents indicated they had not undertaken any training, a similar proportion to that found in the English study [8]. However, in the English study 80% were either 'satisfied' or 'very satisfied' with the training provided, compared with only half in the New Zealand study. NEST aims to provide all participants with training, as a bare minimum a NEP Retailer Manual, introductory pamphlet and an opportunity to view the NEP Pharmacy Training Video at a time that is acceptable for the NEST Coordinator and the pharmacy staff. In the year preceding this survey, NEST coordinators had replaced old manuals with an updated version of the Retailer Manual. Thus all outlets should have a copy of the Retailer Manual and the dispensing protocols (devised by the Pharmaceutical Society in conjunction with the NEP national office) and it is important that all providers are aware of their location in the event of an incident such as a needle-stick injuries requiring adherence to protocols. The fact that around 10% of our respondents believed they had no Retailer manual needs further exploration.

Training needs to be developed which is appropriate and available to staff as well as pharmacists and pharmacy owners. The results indicate that very few pharmacies restrict provision of needle exchange services to the pharmacist only. Furthermore, the attitude of non-pharmacist staff was identified by almost 90% of respondents as being a factor that makes it easier for clients to ask for help. This would indicate a number of areas for developing non-pharmacist staff training. Research indicates that non-pharmacist staff do not receive training – two fifths of the South East England study stated that their staff had not received training [8] and the development of staff training was recommended. In another study of non-pharmacist staff attitudes towards the provision of services for problem drug users, the authors noted that only 5% had attended training, and over one third indicated they wanted further training, in particular in areas such as managing difficult incidents, what is drug misuse, methadone and needle exchange [11]. Whilst it is NEST practise to organise a training session in which as many of the workers at the pharmacy outlet can participate, it is often difficult to arrange such events at convenient times and locations, and therefore such training provides challenges that may need to be overcome with more inventive use of resources such as e-learning.

Support from NEST was another area where improvement might be needed – almost 5% indicated that support from co-ordinators was not available and almost 30% of respondents considered it to be poor. This is interesting considering that the NEST Van Coordinators visit every pharmacy on an eight weekly cycle (some high volume pharmacy outlets are four-weekly), although this may not be considered as 'support'. In addition, the outlet is often phoned in advance to request if there are any issues or training needed and if they need any material. Discrepancies between pharmacists' opinions and NEST intentions may relate to the 'type' of support that pharmacists feel they need, and further work by NEST to accommodate these needs is currently underway.

The study highlighted a number of issues that may prove to be difficult for non-pharmacist staff and pharmacists to address. Shoplifting and intoxicated clients were a relatively common occurrence, and both require staff to be able to handle potentially challenging situations. Furthermore, the issue of the provision of needle exchange and methadone dispensing services can provide ethical dilemmas for many pharmacists, especially when their methadone patient is also using the needle exchange. In practical terms though, few pharmacists refused to supply injecting equipment to such clients.

Another potential problem is when clients do not have enough money to pay for their injecting equipment. Almost one third of pharmacies had a policy of 'no credit under any circumstances' and a further fifth decided on a case-by-case basis. Since the study was undertaken, a free 'one-for-one' service has been made available to clients so they are able obtain a free 'injecting unit' for every used unit returned. Whilst this may cut down on issues around credit, it may also provoke problems when a client has no used equipment with them, but has previously obtained a free syringe and has no money. Currently, community pharmacists self-remunerate through profits on sales of injecting equipment, and the remuneration of pharmacists for service provision may become an issue with the introduction of a new free one-for-one service to New Zealand's needle exchange programme. (NB: pharmacy outlets will take part in this type of service delivery on a voluntary basis only).

Other areas of concern for pharmacists were returns rates of used injecting equipment. A recent audit of returns rates by NEST in 2003 found that exactly 50% of pharmacy outlets had returns of used equipment, although this was often in very low volumes (personal communication). However, a number of points need to be raised here. First, clients may be returning their equipment to stand-alone needle exchange agencies, and a recent NEST audit further supports this (personal communication). In addition, even if not retuned to a participating agency, research from the UK indicates that the majority of clients dispose of their equipment safely and responsibly, for example using personal sharps containers, and throwing them away as part of normal waste [12]. Whether this is the case in New Zealand remains to be explored and future research needs to be conducted with clients around disposal of used injecting equipment.

One simple method which may be employed to improve returns is simply to strongly encourage it; in the English study there was a significant association between strongly encouraging returns and having a higher returns rate [8]. It is anticipated that the upcoming introduction of free one-for-one service in New Zealand will significantly improve the rate of returns to pharmacy outlets as those bringing in their used equipment will be offered the new injecting equipment for free.

Finally, many respondents in the study believed that their other customers were unaware of the needle exchange scheme, and a study of pharmacy customers in Scotland seems to support this [13]. The study further reinforced the idea that customers feel favourably towards needle exchange, understanding the context of harm reduction.

## Conclusion

Needle exchange services in New Zealand have been operational since the late 1980s and this latest survey indicates a healthy and active programme. Surveys of populations of community pharmacists have identified reasons why non-needle exchange pharmacies choose not to engage in service provision, and cite reasons such as lack of time and space, concerns about client behaviour the impact on their business [2]. However, this study found that serious problems such as violence were rare occurrences, and whilst other problems such as shoplifting and disruption by clients were more common, they had not dissuaded pharmacists from staying in the programme – the mean length of time as a needle exchange pharmacist was six years. This is further corroborated by Sheridan et al [8]. It would appear from the activity of these outlets that they are meeting a need, and are an important part of New Zealand's harm reduction response to problem drug use and the prevention of the spread of blood borne viruses.

## Competing interests

CH is national manager of NENZ/NEST

## Authors' contributions

JS designed and managed the study, analysed the data and wrote the paper. NG and AS sent out questionnaires, conducted telephone interviews, entered data into SPSS, undertook preliminary analysis and were involved in editing of the paper. CH provided peer review, drafting of the questionnaire, support for the process including the questionnaire pilot and review, and was involved in editing the paper.
